# An Integrated Deep Learning Model with EfficientNet and ResNet for Accurate Multi-Class Skin Disease Classification

**DOI:** 10.3390/diagnostics15050551

**Published:** 2025-02-25

**Authors:** Madallah Alruwaili, Mahmood Mohamed

**Affiliations:** 1 Department of Computer Engineering and Networks, College of Computer and Information Sciences, Jouf University, Sakaka 72388, Aljouf, Saudi Arabia; 2 Department of Information Systems and Technology, Faculty of Graduate Studies for Statistical Research, Cairo University, Giza 12613, Egypt; mahmoodissr@cu.edu.eg

**Keywords:** fusion-based deep learning, EfficientNet, ResNet, skin disease classification, multi-class classification, dermatological image analysis

## Abstract

**Background:** Medical diagnosis for skin diseases, including leukemia, early skin cancer, benign neoplasms, and alternative disorders, becomes difficult because of external variations among groups of patients. A research goal is to create a fusion-level deep learning model that improves stability and skin disease classification performance. **Methods:** The model design merges three convolutional neural networks (CNNs): EfficientNet-B0, EfficientNet-B2, and ResNet50, which operate independently under distinct branches. The neural network model uses its capability to extract detailed features from multiple strong architectures to reach accurate results along with tight classification precision. A fusion mechanism completes its operation by transmitting extracted features to dense and dropout layers for generalization and reduced dimensionality. Analyses for this research utilized the 27,153-image Kaggle Skin Diseases Image Dataset, which distributed testing materials into training (80%), validation (10%), and testing (10%) portions for ten skin disorder classes. **Results:** Evaluation of the proposed model revealed 99.14% accuracy together with excellent precision, recall, and F1-score metrics. **Conclusions:** The proposed deep learning approach demonstrates strong potential as a starting point for dermatological diagnosis automation since it shows promise for clinical use in skin disease classification.

## 1. Introduction

Skin diseases refer to a subgroup of diseases that affect the skin and thus are not only diverse but also pose serious problems in the management of health care for people of all ages and from all over the world [1]. Recent developments of deep learning techniques for medical image diagnosis apply enhanced chances to increase the diagnosis and classification strategies of dermatological diseases that can help to reform the clinical approaches of skin disorders for the better health and comfort of the patients [2]. Such skin disease features indicate a high demand for accurate and time-saving diagnostic methods, including more frequent pathologies, such as eczema and acne, and severe conditions, like psoriasis and cutaneous lymphoma [3]. However, there is still the problem of inequality in the availability of specialized dermatological services, which leads to several delayed diagnoses and ways of dealing with illnesses [4]. Timely diagnosis is an important aspect in cutting down on death and enhancing treatment plans, especially with the area of focus being melanoma, which has very low survival rates if diagnosed late. However, referrals to dermatological specialists and dermatological technologies are frequently unavailable for patients living in rural or underdeveloped regions, and this is why effective automated diagnosis systems are called for [5]. Recent improvements in deep learning, especially convolutional neural networks (CNNs), have been successfully applied in medical image diagnosis and detection with high automation and accuracy. Currently, there are several categories of state-of-the-art pre-trained CNNs, including EfficientNet [6], ResNet [7], and DenseNet [8], among others, which have proven to provide optimum results when applied to skin lesion classification. Most of these models use transfer learning to learn features that are then used in accurate predictions for medical images without the need for large datasets that are time-consuming to annotate. However, discriminative intra-class variability and inter-class similarities may be an issue for a single CNN in dermatological imaging. Several studies have attempted to solve this problem of skin disease classification with deep learning methods. For instance, Esteva et al. [9] re-tuned an InceptionV3 network and obtained dermatologist-level performance with their study on a large dataset of skin images. Likewise, Tschandl et al. [10] trained several CNNs to diagnose several skin diseases, and they appear to be effective. Nevertheless, these approaches mainly addressed the problem of a single model or homogeneous ensemble, and this can cause some drawbacks when handling imbalanced or noisy databases. Such challenges can easily be solved using a fusion-based method that merges the strengths of the various models. Meštrović et al. [11] investigated the mutual relationship of urinary tract infection development between the gut and vaginal and urinary microbiomes to develop new risk assessment tools and therapeutic approaches. This research examines microbial disruptions and their consequences for avoiding UTIs and treating existing infections. Mujahid et al. [12] investigated the detection of pneumonia in X-ray images through deep learning models, which include Inception-V3, VGG16, and ResNet50. According to experimental findings, the combination of Inception-V3 with the CNN ensemble reached 99.73% in recall and 99.29% in accuracy, which shows promising potential for pneumonia diagnosis. Bordin et al. [13] evaluated diagnostic procedures to detect Helicobacter pylori by dividing them into invasive and non-invasive methods. The combination of endoscopic imaging progress with artificial intelligence boosts real-time diagnosis as the urea breath test alongside the stool antigen test functions as the main diagnostic method for Helicobacter pylori detection. Antibiotic resistance assessment requires the combination of molecular methods together with gastric biopsy cultures. Thomas et al. [14] examined how bacterial populations in the mouth affect both oral infections and chronic systemic health conditions, including caries and periodontal disorders, as well as systemic problems. The authors introduced innovative diagnostic instruments with therapeutic methods based on bacterial recolonization and host modulation therapy, which supports the importance of combined oral–general health professional cooperation.

One of the challenges encountered in this research is class imbalance, especially in skin disease datasets, where some classes have much less data than others, e.g., melanoma is much less represented than benign conditions [15]. Due to the skewed nature, these traditional deep learning models are trained in a way to favor major classes, and hence the performance metrics are off on the minority of classes. As discussed above, the latest studies [16] tried to address this problem with data augmentation and class-weighted loss functions. However, the problem of achieving a balanced overall classification performance has not yet been solved, which requires more effective approaches. Müller et al. [17] have presented an integration technique, including stacking and augmenting, that has been shown to hold great promise in improving the medical image classification systems’ reliability and precision. Li et al. [18] introduced a multimodal medical image fusion approach combining CNN and supervised learning to address limitations in traditional single-image fusion methods. The proposed method enhances fusion quality, detail clarity, and efficiency, demonstrating state-of-the-art performance across various evaluation metrics. Although these fusion techniques appear to achieve higher accuracy, the regularization methods like dropout and batch normalization for improving the generalization performance were not very well incorporated.

In this research, we put forward a fusion-based deep learning model of skin disease classification based on the combination of EfficientNet-B2, EfficientNet-B0, and ResNet50 architectures. These pre-trained models are then employed to extract static and highly informative features of skin disease images. The extracted features are normalized using batch normalization to improve the learning process. Combining these features, the model receives different feature representations of the features, which solves the problem of variations within one class and similarities between different classes. The proposed model includes several dense and dropout layers to better visualize the feature maps and cut down on overfitting. Dropout, on the other hand, helps prevent the model from over-relying on some of the features to improve the overall generalization of the model. These strategies work in conjunction with our proposed model to handle imbalanced datasets with better accuracy in minority classes. This kind of fusion-based approach serves as the solution to all the above-mentioned previous studies, which appeared to use only a single piece of architecture or, at best, did not use complex regularization methods.

Thus, our work contributes to the area by bridging certain known gaps in skin disease classification. The proposed fusion model is superior to a single-model-based approach in that it capitalizes on more than one pre-trained architecture and their complementary qualities to improve outcomes. Furthermore, we also use batch normalization and dropout techniques to reduce overfitting and handle class imbalance problems. A detailed experimental evaluation validates the proposed model on a standard skin disease dataset used in previous studies and shows that it outperforms existing techniques in terms of accuracy. The key contributions of this article are as follows:

1. Propose a fusion-based deep learning model combining EfficientNet-B2, EfficientNet-B0, and ResNet50 for accurate skin disease classification.
2. A feature fusion strategy with batch normalization and dropout layers is introduced to improve generalization and robustness.
3. The proposed model effectively addresses class imbalance and enhances minority class predictions.
4. Comprehensive evaluations on a benchmark skin disease dataset validate the model’s superiority over existing approaches.

The rest of the paper, Section 2, describes the literature review of skin disease classification; after that, the material and methodology are described, and the types of methods used in our study are presented in Section 3. Then, in Section 4, the results and discussions of our work are presented. In addition, Section 5 contains the conclusion of the study.

## 2. Literature Review

Skin disease classification and deep learning approaches have been studied in recent years with high efficiency. Significant to deep learning in dermatology, Esteva et al. [9] used the InceptionV3 architecture to do so. Even though the single-CNN model’s results were as accurate as those of a dermatologist, this design choice may not hold up well in the real world. Likewise, Tschandl et al. [10] applied CNN ensembles, but some issues that turned up included computational overhead and overtraining. In [19], the authors extend their work on deploying advanced deep learning approaches: Vision Transformer (ViT) and convolutional neural networks (CNNs) to detect melanoma. With increased rates of skin cancer and detection of melanoma essential to increase patient survival, the study assesses the performance of these models using a dermoscopic image dataset. The findings also reveal that the pre-trained Vision Transformers yielded an impressive diagnostic accuracy of 97.97%.

Venkata et al. [20] proposed a wrapper feature selection approach based on the dragonfly algorithm (DFA) to select the best feature subsets for skin cancer classification. The researchers combined DFA, where the population of dragonflies has been classified according to their fitness values and brought about the required modification to the positions of dragonflies. In this work, two CNN models were trained using EfficientNet-B2 and VGG19 models and applied data augmentation to both labeled and unlabeled datasets. The framework EfficientNet-B2 yielded excellent performance, specifically in obtaining an overall accuracy of approximately 89.55% on the test set for the classification of some types of skin diseases. It has the potential for use in other dermatological conditions and for other tasks such as psoriasis area and severity index (PASI) scoring, for which this study offers important insights for severity measures and the development of skin disease diagnosis and classification. The authors in [21] focused on deep learning for image analysis on skin disease diagnosis. Therefore, the idea of using skin disease identification brings the consideration of personalization into a potentially more workable reality, with disparate data sources and adherence to ethical AI practices. The authors in [22] described multiple skin lesion classification from dermoscopic images using a shallow model Transformer module (TM) and self-attention unit (SAU) integrated with an efficient CNN model. The proposed lightweight architecture suggests that CNN-based feature generation simplification is possible; simultaneously, the degree of accuracy preservation with dermoscopy images (DIs) and complex textures is possible. Strong performance on the ISIC-2019 and PH2 datasets demonstrates the cross-dataset transferability of the approach via experiments with large sets of other unseen DIs. In the current work, cross-fusion approaches are used to handle dermoscopy images. Also, in [23], the authors presented a new skin disease classification model that utilizes leading-edge deep learning as the key methodology to enhance performance. Based on the MobileNet-V2 architecture, it introduces squeeze-and-excitation networks, atrous spatial pyramid pooling, and the channel attention mechanism. Trained on diverse datasets, including PH2, Skin Cancer MNIST: HAM10000, DermNet, and Skin Cancer ISIC are used as datasets; resizing and mean subtraction normalization are applied. Here, the MobileNet-V2 backbone extracts hierarchical features, whereas ASPP combines multi-scale contextual information to produce a feature map. A study on self-attention-based mechanisms promotes the extraction of inter-channel relationships and contextual information, positively affecting feature discrimination. The model yields an accuracy of 98.6%. Moreover, in [24], the author proposed ResNet50-LSTM, a combination of the ResNet50 deep model and the LSTM classification model, to overcome the shortcomings of existing models. The deep networks are cascaded with the transfer learning technique, which handles the sequential data and captures the structural content of the lesion textures. The findings showed that the third case of ResNet50-LSTM-TL had stunning accuracy, higher than 99.09%, making it even more potent than other deep learning models in detecting different skin cancers.

Although the work done in recent years has enhanced the accuracy and effectiveness of deep learning technology in classifying skin diseases, there are still some drawbacks. Most research considers binary or few categories of classification and so does not offer broad solutions for classifying skin diseases concurrently. For instance, current models that are presented in the literature fail to predict all ten standard labels of skin diseases at the same time; therefore, they cannot be implemented in real-life clinical practice. The proposed model fulfills the need for better-automated skin disease classification since InceptionNet-v3 and other existing deep-learning models prove insufficient. Traditional approaches fall short of accurate diagnosis when used for skin condition identification since various skin diseases share visual patterns that produce diagnostic errors through misclassification errors. A proposed model improves classification results through state-of-the-art feature extraction processes that minimize misclassification errors while increasing general performance. An optimized deep learning architecture powerfully differentiates different dermatological conditions, which makes the model beneficial for clinical decision support applications alongside telemedicine systems.

## 3. Materials and Methods

In this section, we describe the models used in our proposed model, such as ResNet50, EfficientNetB0, and B2, and the rationale behind their selection and fusion. ResNet-50 demonstrated choice as a feature extractor because its deep residual connections address gradient vanishing problems, thus resulting in effective feature transmission. The combination of EfficientNet variants B0 and B2 needed integration because they demonstrated high parameter efficiency together with a balanced trade-off between network depth and width while running fewer computations. The proposed fusion model selects strengths from multiple network architectures that will use different feature structures to boost classification results. CancerNet is expected to deliver improved overall performance by uniting standard models because it improves both generalization capabilities and misclassification resistance when classifying visually similar skin diseases.

### 3.1. ResNet50

**Figure 1:**
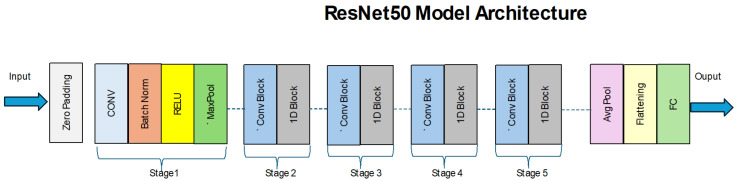
ResNet50 model architecture.

There is a CNN model called ResNet50, which stands for Residual Network-50, aimed at resolving difficulties with training very deep neural networks, mostly the vanishing gradient drawbacks. As proposed by He et al. [7], ResNet50, as shown in Figure 1, has 50 layers and is based on what is known as residual learning with shortcut connections. These shortcut connections allow the network to jump over one or more of them and perform an identity function, thereby making learning much easier. Unlike MobileNet, ResNet50 learns not direct mappings but residual functions, which should make the backpropagation step smoother. This enables the establishment of deeper models as far as the innovation helps in training the same without compromising on its performance, which is much different from the original architectures like VGG16 and AlexNet.

Figure 1 depicts ResNet-50’s architectural design, adopting deep residual learning to extract features with added protection against gradient vanishing. The initial architecture starts with an input layer, and it completes zero padding for spatial dimension protection. The first stage contains Conv layers for feature extraction, after which Batch Norm helps stabilize training before the model applies ReLU and Max Pool for down-sampled feature maps. The architecture includes stages 2 through 5 with convolution combined with identity blocks, which apply learnable filters within convolutional blocks but let gradient flow pass through shortcut connections. The framework implements an average pooling (average pool) together with a flattening layer to complete its operation. The average pooling functionality helps decrease dimensions before the final layer transforms feature maps into one-dimensional vectors. The output predictions emerge from the last fully connected layer, which concludes the classification process. The systematic design of ResNet-50 enables both performance in organizing hierarchical models and operational efficiency.

ResNet50 has residual blocks where 1 × 1, 3 × 3, and again 1 × 1 layers are used for the reduction of dimensionality, feature extraction, and expansion, respectively. In the same manner, ResNet50 decreases computational costs and Cal energies via bottleneck designs without compromising the network’s ability to contain sufficient representational capacities. Because of this learning of hierarchical features, CNNs have been adopted in image classification chores, performing impressively well on benchmark image datasets, such as ImageNet. Besides the classification context, ResNet50 has also been used in many other computer vision tasks, such as object detection, image segmentation, and medical image analysis [25,26]. Due to its modularity and efficiency, the authors mentioned above noted that it has become the base for many other derived architectures, primarily ResNet and DenseNet, due to its residual learning capability [8]. Nonetheless, ResNet50 has its drawbacks: Although residual learning helps to reduce gradient problems, the depth of the network poses a problem of computational and memory overhead, especially in environments where such resources are scarce. Fine-tuning deep networks such as ResNet50 may be very sensitive and compelled by optimization considerations and hyperparameters’ settings. However, on balance, ResNet50 is the most important accomplishment in deep learning because it learns deep networks well and generalizes well in many contexts. It has paved the way for the improvement of subsequent deep learning frameworks in computer vision, which marks the study as a strong foundation in modern computer vision [7,8].

### 3.2. Efficient Net

**Figure 2:**
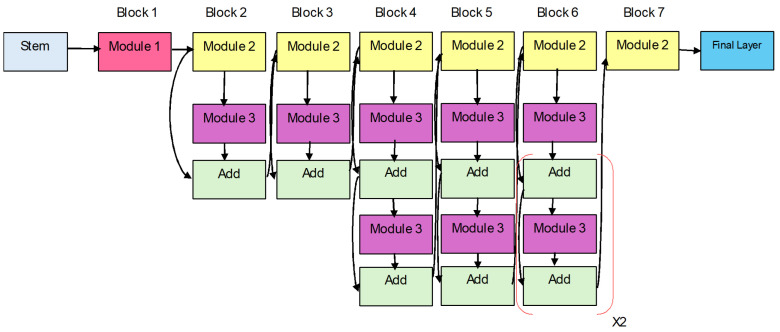
EfficientNet-B0 Model Architecture.

Tan and Le [6] proposed a novel CNN called EfficientNet, improving both efficiency and accuracy through a proper scaling of depth, width, and resolution of the input data. As opposed to previous works, EfficientNet applies a compound scaling factor to these dimensions so that all are positively scaled while at the same time maximizing computational complexity. With EfficientNet-B0 as the baseline model, launched from neural architecture search (NAS), which maximizes the architecture density while providing extremely high efficiency. Even though it has merely 5.3 M parameters, EfficientNet-B0, as shown in Figure 2, provides high results in image classification tasks, is energy efficient, and even surpasses heavy-hitter models, such as ResNet50 on the ImageNet dataset. It comprises mobile inverted bottleneck convolution (MBConv) blocks, depth-wise convolutions, and SWISH activation functions, all of which enable the model to pick fine-grained feature representation with much less computational cost.

The EfficientNetB0 design shows the network architecture used for efficient and scalable image classification duties, as shown in Figure 2. As the base model of the EfficientNet family, EfficientNetB0 applies a compound scaling methodology for equal scaling of network depth, width, and resolution. The model design permits both high-accuracy performance and efficient operation. Modularity refers to the network design, which exhibits multiple numbered components extending from Module 1 through Module 584. The modular approach in the network design represents individual network elements, such as convolutional layers or activation functions, as well as pooling layers. The systematic and hierarchical EfficientNetB0 architecture achieves optimal performance–resource utilization through its structure/modules numbered from 1 to 584.

EfficientNet-B2 is developed from the base EfficientNet-B0 model using compound scaling, which scales network depth, width, and input resolution in a proportional manner. More precisely, B2 enlarges the input image resolution to 260 × 260, enlarges the number of channels in the convolutional block, and increases the depth of the model in comparison with B0. Such enhancements enable EfficientNet-B2 to better encode finer-scale spatial patterns yet be efficient. EfficientNet-B2 has fewer parameters than B0, with approximately 9.1 million; however, it has slightly better performance in terms of accuracy, so it can be preferred for tasks that require finer features, such as medical image classification and fine-grained classification [27]. Nevertheless, B2 enlarges its scale while remaining computationally efficient, thanks to its efficient layout design and the selection of dimension scaling.

**Figure 3:**
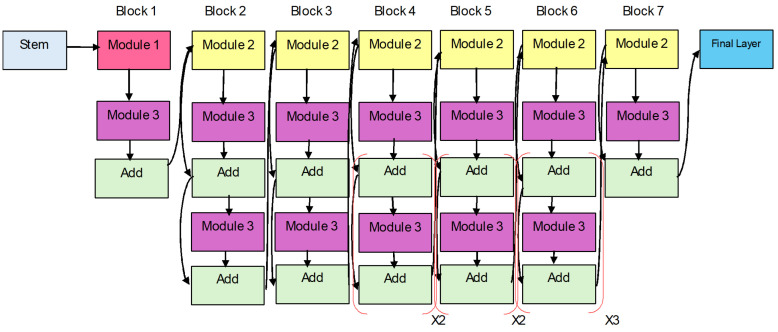
EfficientNet-B2 model architecture.

Figure 3 depicts the EfficientNet-B2 architecture, which functions as a convolutional neural network platform optimized for scalable image classification. A modular design structure is evident through the large number of numbered modules ranging from Module 1 up to Module 586. Every separate module matches a distinct part of the network, including convolutional modules and activation functions alongside pooling blocks. The sequential arrangement of these modules explains how EfficientNetB2 follows a systematic hierarchy that ensures optimal performance alongside resource efficiency in deep learning tasks. A modification of EfficientNetB0 implements expanded scaling parameters to achieve better precision and resistance in this model.

Recent members of the EfficientNet family, namely B0 and B2, have outperformed their counterparts, leveling high scores within numerous computer vision tasks, notably ImageNet. Due to their highly accurate results with low computational complexities, they are used in a wide range of applications, from mobile applications to large-scale image recognition [28]. However, due to compound scaling, the growth of model performance is limited to the baseline architecture, and any additional increase must be obtained through computationally intensive NASs. Still, EfficientNet-B0 and B2 provide insight into effective model scaling and have succeeded in efficient deep learning solutions in various domains [6,29].

In the proposed model, we select the EfficientNetB0 and EfficientNetB2 models because they achieve a perfect trade-off between resource efficiency and performance, which makes them ideal for various applications that work within limited computing capacity. The two EfficientNet versions establish a foundation through EfficientNetB0, which provides the compact architectural design with compound scaling as well as the enhanced accuracy capabilities of EfficientNetB2 without additional computational complexity. The orientation stems from requirements to use accuracy-strong and deployment-friendly models for practical use, yet higher variants B7 and B8 demonstrate insufficient performance despite requiring more resources. The implementation of B0 and B2 allows for an applicable solution that provides both versatility and computational efficiency for different operational domains.

### 3.3. Proposed Fusion Model

**Figure 4:**
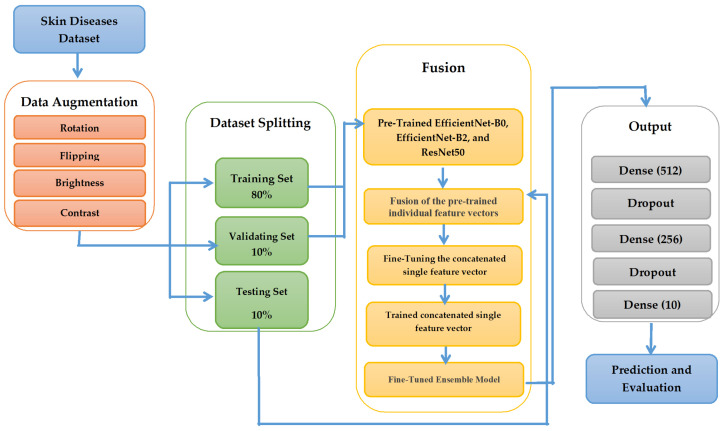
Proposed fusion model.

The proposed fusion model for skin disease classification illustrated in Figure 4 operates as an architecture for automated skin disease classification. The proposed approach employs three convolutional neural networks named EfficientNet-B0, EfficientNet-B2, and ResNet50 that extract multiple feature patterns from medical images. Each CNN performs separate analyses on the input images while building deep hierarchical features until reaching a holistic representation of the features. The fused features move through fully connected layers until the SoftMax layer makes a final classification among the ten skin disease categories.

#### 3.3.1. Data Preprocessing

The data processing stage starts by applying preprocessing and augmentation techniques to the skin disease dataset before continuing to the training process. Data augmentation adopts techniques such as rotation, flipping, brightness, and contrast adjustment. Additionally, rotation serves to provide real-world simulation effects alongside flipping mechanisms that perform horizontal and vertical transformations for increasing data variety. Adjustments in brightness levels and contrast values enable the model to understand different levels of illumination conditions more effectively. The group of augmentation techniques works together to improve how the model detects skin diseases across different setting conditions. Data augmentation enables the subdivision of the dataset into three parts, where 80% serves for training purposes and 10% functions for validation, while the remaining 10% serves for testing purposes. The model optimizes its parameters during training with the data from the training set, and the validation set confirms hyperparameter adjustments and stops overfitting scenarios. The testing set provides the final model evaluation, so performance metrics exactly gauge actual real-world generalization performance.

#### 3.3.2. Feature Fusion and Classification

The main component of the model combines three pre-trained powerful deep learning representatives known as EfficientNet-B0, EfficientNet-B2, and ResNet50 for feature extraction through fusion. The input images undergo high-level feature representation processing by each model to detect various elements of skin disease patterns. The classification procedure combines model-generated feature vectors through concatenation to attain an enriched feature set that receives additional optimization for classification purposes. Using this feature fusion technique enhances the compilation of robust features compared to standalone model usage. The classification process begins after the fused feature vector goes through a dense layer sequence that contains numerous fully connected blocks. The final classification head contains three linearly dense layers with 512, then 256, and finally 10 that use SoftMax activation. Between each dense layer exists a Dropout layer. During training, the dropout layers work as a regularization tool that creates better generalization through random neuron deactivation. Finally, the output layer performs categorical classification aimed at predicting the nature of skin disease. The SoftMax activation function gives probability scores for each class, and then the model can predict the most probable label of the input images. The types of skin diseases to be classified in the proposed fusion model include eczema, melanoma, atopic dermatitis, basal cell carcinoma (BCC), melanocytic nevi (NV), and benign keratosis-like lesions (BKL).

#### 3.3.3. Evaluation Metrics

Multiple evaluation metrics determine the effectiveness assessment of the proposed model. The main measure of classification accuracy consists of accuracy. The evaluation of model performance includes calculations of precision, recall, and F1-score to identify how well the model distinguishes between various skin disease classes while maintaining sensitivity and specificity. Assessment through the area under the receiver operating characteristic (AUC-ROC) curve analysis allows measurement of model discrimination power between classes across different classification threshold points. The proposed model reaches better classification accuracy together with improved generalization ability through its integration of multiple pre-trained CNNs and optimized feature fusion techniques. The model’s reproducibility stems from structured preprocessing procedures along with available scripts and systematic testing methods that allow real-world skin disease diagnosis applications.

## 4. Experimental Results and Discussion

The section presents an in-depth description of the dataset while also providing performance mathematical equations followed by experimental results and a state-of-the-art comparison discussion.

### 4.1. Dataset Description

The Skin Diseases Image Dataset is available on Kaggle [30] and provides a vast collection of original, visually identified skin images intended for a range of dermatological disease classifications. The dataset is 5.58 GB large. It has 10 different labels containing different skin diseases and 27,153 images of clinical images that demonstrate variations in skin color and patterns and range from mild forms of skin diseases to severe. It also enables reliable model formulation and evaluation by catering to the diverse characteristics of the dataset. The proposed model was accessed through TensorFlow and Keras tools on an NVIDIA GPU device that provided quick model training plus evaluation processes. An Adam optimizer initiated at a 0.0001 learning rate powered all operations and hidden layers, using ReLU activation before the Softmax operation generated class probabilities. The dataset was divided into training (80%), validation (10%), and testing (10%) splits, which remained constant for all models during the comparison process. To establish the accuracy of our findings, we conducted 5-fold cross-validation, which strengthened performance assessment while reducing potential biases in the dataset.

The distribution of skin conditions contains 10 distinct categories that show their occurrence numbers in the presented Table 1. The research reveals that 1677 persons suffer from eczema, and 3140 individuals experience melanoma. The record shows that atopic dermatitis affects 1257 cases, whereas basal cell carcinoma (BCC) has 3323 cases. The dermatological condition classification has 7970 cases of melanocytic nevi (NV) as the most common and 2079 cases of benign keratosis-like lesion (BKL) as the second most common. The reports for psoriasis and lichen planus, along with related conditions, represent 2055 cases and seborrheic keratoses with other benign tumors represent 1847 cases. Of the recorded skin conditions, Tinea ringworm, Candidiasis, and other fungal infections total 1702 cases, while warts, molluscum, and other viral infections amount to 2103 cases. The dataset demonstrates how different skin-related diseases occur throughout their specified areas.

The analysis uses the hyperparameters in Table 2 to attain peak training outcomes. Because of its effectiveness on big datasets and generalization capabilities, the stochastic gradient descent (SGD) optimizer was the choice. The learning rate was set to 0.0001 to achieve gradual convergence and reduce the possibility of seeking past the ideal solution. The models were trained through the suitable multi-class classification loss function known as categorical cross-entropy. The model performance evaluation used accuracy as the main metric. The model used early stopping through validation loss monitoring to prevent overfitting using a patience threshold set to 20 epochs. The restore best weights capability was enabled to save the model parameters that exhibited peak performance for ultimate assessment.

Several approaches were used to handle the class imbalance problem in the image dataset by the proposed system. The minority classes benefited from data augmentation procedures that produced synthetic data by transforming real images with rotation effects and flipping operations in addition to brightness and contrast transformations. The combination of EfficientNet-B0, EfficientNet-B2, and ResNet50 allowed the model to extract comprehensive features from skin disease images, thereby reducing reliance on the majority class data. A weighting mechanism was included in the model’s loss function structure to prioritize the training of minority classes, therefore achieving balanced performance across all diagnosis categories. By applying these combined strategies, the model gained effective control over the class imbalance, which resulted in strong classification outcomes.

### 4.2. Performance Metrics

The evaluation of each classifier depended on accuracy levels while also using classification reports alongside ROC curves. The accuracy evaluation relied on dividing correctly classified instances by the total instances present. The evaluation process provided initial precision, recall (detection rate), and F1 scores for each test set class in classification reports. The comparison of model effectiveness through different classification thresholds employed ROC curves along with their area under the curve (AUC) evaluation.

(1) $$\mathrm{Accuracy}=\frac{TP+TN}{TP+FP+TN+FN}$$

(2) $$\mathrm{Precision}=\frac{TP}{TP+FP}$$

(3) $$\mathrm{Recall}=\frac{TP}{TP+FN}$$

(4) $$F1-\mathrm{measure}=\frac{2TP}{2TP+FP+FN}$$

The model performs assessment with four categories, including true positives (TPs), true negatives (TNs), false positives (FPs), and false negatives (FNs).

### 4.3. Results

Here, we report the comparative results of the deep learning models alongside the proposed model, such as EfficientNet-B2, ResNet101V2, MobileNet-v3, and InceptionNet-v3. To evaluate how well these models are for dermatological disease classification, they were examined using the Skin Diseases Image Dataset. Their performances were evaluated with the help of accuracy, precision, recall, and F1-score. The experiments for pre-trained model comparison depended on standardized execution procedures. The comparison involved maintaining consistent dataset partitioning methods along with preprocessing requirements while using the stochastic gradient descent optimizer and identical learning rates and batch sizes across all experimented models. The training duration changed according to early stopping, which used validation loss to stop training before overfitting occurred. The training duration for EfficientNet-B2 models needed only 70+ epochs for convergence, yet ResNet101V2 required 20+ epochs to achieve maximum performance, MobileNet-v3 required 60 epochs for convergence, InceptionNet-v3 required 100 epochs for convergence, and the final proposed model required 20 epochs for convergence. We explicitly added in the manuscript that all models achieved convergence under uniform experimental conditions regardless of their various epoch numbers.

Table 3 shows the classification report of testing the Skin Diseases Image Dataset for five models, including EfficientNet-B2, ResNet101V2, MobileNet-V3, InceptionNet-V3, and the proposed model. The execution time of 100 seconds represents the fastest performance of MobileNet-V3 along with a fair precision and recall score of 0.85 that also produces comparable F1-score and accuracy levels. The computational time was 170 seconds for ResNet101V2 while it delivered a performance of 0.80 across metrics. The InceptionNet-V3 model delivered moderate accuracy for skin disease diagnosis through 150 seconds of execution time, together with precision at 0.82 and F1-score and accuracy at 0.81. The proposed model delivered superior performance compared to all other models tested by reaching 99.14% across all assessment indicators within 200 seconds of execution time. Its diagnostic capabilities for identifying 10 skin disease conditions make the proposed model a powerful diagnostic instrument even though it requires slightly more computer processing time.

**Figure 5:**
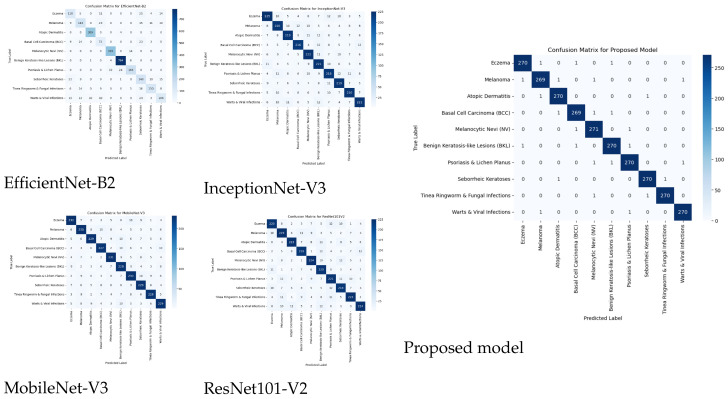
Confusion matrices.

Figure 5 shows the performance evaluation through confusion matrices of four deep learning models, including ResNet101V2, MobileNet-V3, and InceptionNet-V3, and the proposed model appears in the figure based on their assessment in a ten-class classification scenario. The classification performance becomes visible in each confusion matrix through its presentation of properly identified and wrongly identified sample counts for different classes. Efficient Net-B2 classification accuracy reaches 84% but enables too many misdiagnoses between disparate categories across all non-diagonal elements. ResNet101V2 confirms moderate diagnostic errors between related dermatological conditions when achieving an 80% overall success rate. The classification capability of MobileNet-V3 reaches 85% accuracy because it shows more correct predictions along its main diagonal. InceptionNet-V3, with an accuracy of 81%, exhibits a slightly higher misclassification rate than MobileNet-V3 but outperforms ResNet101V2. The proposed model delivers exceptional performance by exceeding all baseline models through its 99.14% accuracy rating. The proposed model achieves nearly perfect precision, recall, and F1 score for all classes based on its strong diagonal values and low number of misclassified predictions found in its confusion matrix. The proposed model provides an effective solution for differentiating between dermatological conditions because its performance steadily improves in comparison to other models evaluated.

**Figure 6:**
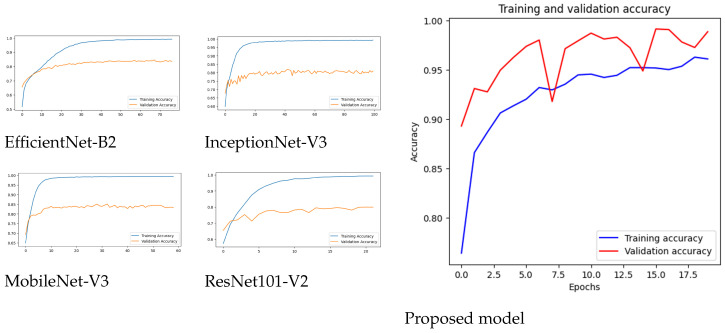
Accuracies for all models.

Figure 6 displays the accuracy changes of five deep learning models, including EfficientNet-B2, ResNet101V2, MobileNet-V3, InceptionNet-V3, and the proposed model, during their training and validation cycles across different epochs. The training accuracy of EfficientNet-B2 steadily rises to indicate 100% success, whereas its validation accuracy stabilizes at 85%, which implies weak model overfitting potential. ResNet101V2 demonstrates solid training accuracy along with validation accuracy that stops below 80%, potentially because of model generalization difficulties. MobileNet-V3 demonstrates fast learning, which results in nearly 85% validation accuracy, as well as stable training–validation accuracy differences. Training accuracy from InceptionNet-V3 achieved near-perfect results, but validation accuracy stagnated at 81%, which indicates steps toward overfitting occurred. A high validation accuracy emerges from the proposed model, which demonstrates minimal fluctuations and suggests better generalization potential. The proposed model demonstrates excellent stability between its training accuracy and validation accuracy, which suggests more robustness together with lower overfitting than the other competing models. The research findings demonstrate that the proposed model outperforms alternative models because it shows better results in complex pattern detection combined with strong generalization capabilities.

**Figure 7:**
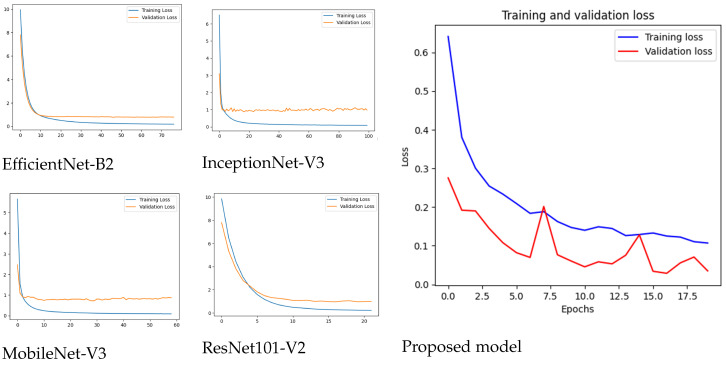
Losses for all models.

Figure 7 shows a comparison of convergence and generalization capacity exists between the training and validation loss curves of five models, including EfficientNet-B2 and ResNet101V2, along with MobileNet-V3, InceptionNet-V3, and the proposed model in a single depiction. The EfficientNet-B2 model demonstrates quick loss decreases during training and displays steady validation loss at a slightly higher level than training loss because of limited model overfitting. ResNet101V2 demonstrates training and validation loss patterns that show a significant gap that signals potential overfitting of the model. The convergence speed of MobileNet-V3 and InceptionNet-V3 enables their training loss to approach zero values as the validation loss stays steady, indicating acceptable but not peak generalizability. A distinct characteristic of the proposed model lies in its stable loss reduction pattern because training loss correlates closely with validation loss while showing excellent generalization capacity. The proposed model demonstrates lower overfitting through its minimal training–validation loss gap; thus, it offers enhanced performance for complex data distributions.

**Figure 8:**
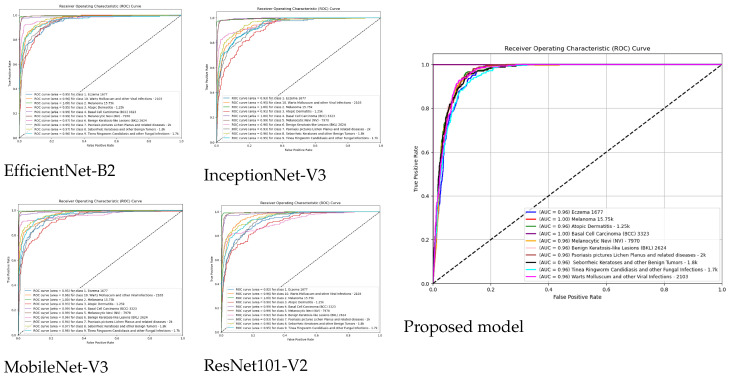
ROC curve for all models.

Figure 8 shows receiver operating characteristic (ROC) curve analysis with performance evaluation of ten distinct classes, including eczema, alongside melanoma and basal cell carcinoma. The representation of the true positive rate (TPR) vs. false positive rate (FPR) trade-offs in specific classes occurs through individual curves alongside area under the curve (AUC) metrics, which assess model distinction capability. The AUC = 1.00 perfect separation results of classes melanoma and basal cell carcinoma are distinguished from other classes such as eczema and warts molluscum, which exhibit strong but sub-perfect performance, with AUC values between 0.93 and 0.99. Random guessing stands as the reference point, while model effectiveness increases when the curves get closer to the top-left area of the graph. High classification accuracy stands out in the figure, even as performance shows varying levels across specified conditions, especially for selected classes.

### 4.4. Discussion

The fusion-based deep learning model, which combined EfficientNet-B0, EfficientNet-B2, and ResNet50, reached excellent results in skin disease classification, with an accuracy of 99.14%. The dual benefit of merging multiple pre-trained architectural frameworks allowed the model to acquire different features, thereby strengthening its ability to perform generalizations across diverse skin conditions. The utilization of single-pretrained EfficientNet-B2 proves effective, but the multi-architecture fusion approach extracts wider features while achieving optimal performance in skin disease classification. The proposed model benefited from additional regularization techniques, which prevented overfitting while maintaining training stability to enhance its total performance.

The proposed model achieves better class imbalance performance than MobileNet-v3 and ResNet101v2. The design of MobileNet-v3 focuses on efficiency, which results in an accuracy level reaching 85%, although this result makes it inadequate for analyzing complex medical datasets needed for skin disease diagnosis. Efforts to use ResNet101v2 resulted in 80% accuracy, yet the model faced challenges when dealing with multiple diseases and similar patterns between them. The proposed fusion model delivers a comprehensive performance improvement through an accuracy of 99.14%. Through fusion, the model benefits from multiple network-learned features to achieve precise identification of skin disease variations.

The combination model demonstrates superior performance compared to Inception and EfficientNet-B2 on challenging multi-class skin disease identification tasks. The distribution–meaning reduction by convolution filters of Inception networks creates a challenge when dealing with intra-class differences in data. EfficientNet-B2 shows superior performance when it comes to both parameter utilization and processing speed, but the accuracy is 84% for detecting multi-class skin diseases. The proposed model merges EfficientNet-B0 with EfficientNet-B2 plus ResNet50 as its components to overcome these hurdles since the combination enhances performance for minority class detection and guarantees improved accuracy in all categories.

Comparative analysis shows the evolution and further developments in skin disease classification methods adopted by deep learning frameworks, as shown in Table 4. The study of Venkata et al. [16] involved the use of the EfficientNet-B2 model on the DermNet NZ Image Library and was 89.55% accurate. While this result is quite impressive, it specializes in identifying simple patterns that need some improvement when it comes to capturing complex skin disease patterns. In contrast, K. Vayadande [17] only adopted regularization techniques in the CNN framework, whereas the datasets were collected from the Kaggle site and obtained a striking accuracy of 98%. This improvement is notable for proving the idea of enhancing the CNN architecture designed for generalization and model robustness. Similarly, Rezaee [18] used capsule networks to enhance feature extraction, with an accuracy of 96.87% on the PH2 dataset, and warned that further improvement may be required to achieve greater levels of accuracy. However, the sophistication of models that have been developed in the recent past, like those by Nirupama [19] and Mavaddati [20], has led to improved benchmarks in skin disease classification. Thus, by using MobileNet-V2 and applying additional modules, such as squeeze-and-excitation networks and atrous spatial pyramid pooling, Nirupama achieved 98.6% accuracy on multiple datasets. To round off, Mavaddati developed a ResNet50-LSTM to increase accuracy by a margin; the repeatability of 99.09% proved this was possible when using both convolutional and sequential models to capture lesion texture and structural content. These include the following smart state-of-the-art techniques that the proposed model outperforms, yielding a maximum accuracy of 99.14% on datasets from Kaggle. This shows that the proposed model can be used in real-world applications since it indicates high classification accuracy while at the same time presenting a workable solution for classification methods useable for various data sizes and applicable in scalable classifications.

## 5. Conclusions

The deep learning model that fuses a classification network with two pre-trained networks shows better results for identifying skin diseases. The proposed model design effectively captures detailed visual characteristics in dermatological images through its multi-stream architecture that implements EfficientNet-B0, EfficientNet-B2, and ResNet50 components. The combination strategy permits these networks to work together using their respective strengths while resolving the unique challenges that single models encounter during both inter-class and intra-class classification. The model delivers exceptional results on the Kaggle Skin Diseases Image Dataset with 99.14% accuracy together with matching precision and recall values and an F1 score of 0.9914, which validates its diagnostic credibility. The model executes medical image analysis with deep learning-based complexity while operating within 200 seconds of execution time. The research results demonstrate how model fusion approaches can boost dermatological image classification procedures. Deep learning establishes itself as a dependable tool for diagnostic support in medical skin disease assessment by achieving nearly flawless performance. These outcomes create standards for medical imaging research that advance the practical applicability of modern deep learning approaches in healthcare.

Several important obstacles must be considered. The quality of data and its variation level determine how well the model functions. The model is now limited to the Kaggle dataset, which contains a consistent group of skin diseases or conditions, but it cannot diagnose any other pathologies, and performance is unknown on other datasets. Training and inference procedures from this system require significant computational power that becomes a major factor for adoption among resource-restricted locations, including mobile devices and rural healthcare services. The model requires external validation with various datasets before it can be used clinically because its effectiveness on external datasets remains untested. Future research must devote efforts to building a larger database that includes various skin disorders, particularly uncommon ones, to extend the model’s generalization abilities. The optimized model design will make it feasible to use this system in real-time diagnostic tools alongside mobile health applications. Enhanced predictions will result from processing multiple types of patient information that include demographic features alongside clinical background records. By implementing the model into clinical decision support systems, healthcare professionals would gain more effective disease diagnosis capabilities. This study positions itself as the foundation for developing future sophisticated dermatological diagnosis tools that will deliver better accessibility and durability through AI technology.

## Figures and Tables

**Table 1:** Distribution of skin disease classes with their respective counts.

Class Name	Count
Eczema	1677
Melanoma	3140
Atopic dermatitis	1257
Basal cell carcinoma (BCC)	3323
Melanocytic nevi (NV)	7970
Benign keratosis-like lesion (BKL)	2079
Psoriasis, lichen planus, and related diseases	2055
Seborrheic keratoses and other benign tumors	1847
Tinea, ringworm, candidiasis, and other fungal infections	1702
Warts, molluscum, and other viral infections	2103

**Table 2:** Hyperparameters for all models.

Hyperparameter	Value
Optimizer	Stochastic Gradient Descent (SGD)
Learning Rate	0.0001
Loss Function	Categorical Cross entropy
Metrics	Accuracy
Monitor	Validation Loss
Patience	20 epochs
Restore Best Weights	TRUE

**Table 3:** Classification report of the testing dataset.

Model	Precision	Recall	F1-Score	Accuracy	Execution Time in Second
EfficientNet-B2	0.84	0.84	0.84	0.84	140
ResNet101V2	0.80	0.80	0.80	0.80	170
MobileNet-v3	0.85	0.85	0.85	0.85	100
InceptionNet-v3	0.82	0.81	0.81	0.81	150
Proposed Model	0.9914	0.9914	0.9914	0.9914	200

**Table 4:** Comparative analysis of the proposed model vs. state-of-the-art methods.

Reference No.	Year	Technique	Collections	Accuracy
Venkata et al. [20]	2024	EfficientNet-B2 (CNN model)	DermNet NZ Image Library	89.55
K. Vayadande [21]	2024	Regularization techniques within the CNN framework	Datasets sourced from Kaggle	98.00
Rezaee [22]	2024	Utilization of capsule networks for improved feature extraction	PH2 datasets	
Nirupama [23]	2024	MobileNet-V2	PH2 dataset, Skin Cancer MNIST: HAM10000 dataset, DermNet. dataset, and Skin Cancer ISIC dataset.	98.6
Mavaddati [24]	2025	ResNet50 and LSTM	Skin Dataset	99.09
Proposed Model	-	ResNet50 and EfficientNet(B0, B2)	Skin Datasets sourced from Kaggle	99.14

## Data Availability

Images dataset available at https://www.kaggle.com/datasets/ismailpromus/skin-diseases-image-dataset, (accessed on 5 January 2025).
